# Sulfonation and Characterization of Tert-Butyl Styrene/Styrene/Isoprene Copolymer and Polypropylene Blends for Blood Compatibility Applications

**DOI:** 10.3390/polym12061351

**Published:** 2020-06-15

**Authors:** Bin-Hong Tsai, Yung-Han Chuang, Chi-Hui Cheng, Jui-Che Lin

**Affiliations:** 1 Department of Chemical Engineering, National Cheng Kung University, Tainan 701, Taiwan; colintsai0324@gmail.com (B.-H.T.); sandy840530@gmail.com (Y.-H.C.); 2 Department of Pediatrics, College of Medicine, Chang Gung University, Chang Gung Memorial Hospital, Taoyuan 333, Taiwan

**Keywords:** sulfonation, elastic styrenic block copolymer, hydrogenation, polypropylene, platelet compatibility

## Abstract

Hydrogenated styrenic block copolymers (HSBCs) have been used in medical tubing for many years due to their high clarity, flexibility, kink resistance, and toughness. However, when it comes to blood storage applications, HSBC compounds’ market has been limited because of their high hydrophobicity, which may trigger platelet adhesion when contacting with blood. HSBC needs to be physically or chemically modified in advance to make it blood compatible; however, HSBC has strong UV/ozone resistance, thermooxidative stability, and excellent processing capability, which increases the difficulty of the chemical modification process as unsaturated dienes has been converted to saturated stable midblocks. Moreover, medical HSBC-containing compounds primarily make up with the non-polar, hydrophobic nature and benign characteristics of other common ingredients (U.S. Pharmacopeia (USP) grades of mineral oil and polypropylene), which complicates the realization of using HSBC-containing compounds in blood-contacting applications, and this explains why few studies had disclosed chemical modification for biocompatibility improvement on HSBC-containing compounds. Sulfonation has been reported as an effective way to improve the material’s blood/platelet compatibility. In this study, hydrogenated tert-butyl styrene (tBS)-styrene-isoprene block copolymers were synthesized and its blends with polypropylene and USP grades of mineral oil were selectively sulfonated by reaction with acetyl sulfate. By controlling the ratio of the hydrogenated tBS-styrene-isoprene block copolymer in the blend, sulfonated films were optimized to demonstrate sufficient physical integrity in water as well as thermal stability, hydrophilicity, and platelet compatibility.

## 1. Introduction

Neat polyvinyl chloride, PVC, is essentially rigid, and, henceforth, it is inevitable that adding plasticizer additives will impart flexible properties. Plasticized PVC is widely used in blood storage and intravenous solution bag applications. Di (2-ethyl hexyl) phthalate, DHEP, a common plasticizer commonly used in PVC blood bags has been proven to be leached out from medical storage bags and would jeopardize the infant fertility and development [1]. To address the potential issues of DEHP, scientists continue to search for alternatives to current polymeric PVC blood bags. The global annual blood bag demand is more than 900 million units and the cost is also a very important factor due to medical equality concern [2]. Therefore, efforts had been put on the exploration of the general five plastics development for such biomedical applications [3,4,5,6]. However, using the general five plastics to replace PVC is challenging due to lack of acceptable flexibility, temperature resistance, and transparency that are potential key factors as a candidate for medical storage bag replacement [7]. Among general five plastics, polypropylene, a simple polymer, is more reactive for having a methyl side group. Some studies have attempted to introduce polar elements, such as sulfonic groups into polypropylene for improving surface hydrophilicity and the ability to modulate the biocompatibility with surrounding tissue when part of an implant [8,9]. However, when it comes to medical storage, the critical disadvantage of polypropylene is low-impact strength at low temperatures. 

In the 1960s, Shell developed an anionic polymerization process for the synthesis of the styrene block copolymer (SBC), Kraton^®^, which has a whole family of styrenic block copolymer for different applications. The benefits of SBC include improved processability and compatibility with polypropylene (PP) for physical property enhancement. Following years of investigation, a series of materials combining Kraton^®^ with PP was found with promising effects of improved property-broadening glass transition temperature below room temperature. This opens several opportunities for using the SBC-PP mixture as a potential candidate for PVC replacement. However, the biocompatibility and cost of SBC-PP blends are still challenges [10]. 

Besides, making thinner SBC films for a variety of useful applications, such as membrane materials and antimicrobial films, was also difficult because the films obtained after heat treatment do not maintain their forms and immediately shrink and deform. Therefore, SBC thin films were prepared as a composite by using PP and wax. These hydrophobic ingredients also put a limit on SBC in biomedical applications. To improve hydrogenated styrenic block copolymers (HSBCs) hydrophilicity without losing their physical integrity, Kraton has developed a controllable sulfonated pentablock styrenic copolymer (s-PBC) commercialized as Nexar^TM^. Functionalization with sulfonic groups in Nexar^TM^ allows this polymer to achieve an excellent balance among various applications, mainly thanks to its block molecular structure and functionalization with sulfonic hydrophilicity and mechanical stability. The unreacted tert-butyl functional end group prohibits the resultant block copolymers from dissolving in water and sufficient mechanical strength of swollen copolymer films was also maintained. This approach provides a new potential of incorporating controllable sulfonate/sulfonic groups into the inert HSBC chemical structure that could improve the hemocompatibility as well as increase the hydrophilicity of biomaterials [11,12].

In this study, multi-block tert-butyl styrene (tBS)/styrene/isoprene block copolymer (tSIS) was synthesized by the polymerization of different ratios of tert-butyl styrene, styrene and isoprene. In order to enhance the thermal stability of tSIS block copolymer, saturated tBS/styrene/isoprene, the tSEPS (tert-butyl styrene/styrene-ethylene-propylene-tert-butyl styrene/styrene), was obtained by the hydrogenation process. This would increase the thermal stability of block copolymer, which can be sterilized under 121 °C [13,14,15,16]. Sulfonation of the tSEPS/PP/mineral oil blended films was executed with acetyl sulfate. The chemical and mechanical characteristics of these polymers as well as the blended films were characterized. Besides, the platelet-contacting properties of the blended films were also analyzed. It was noted that films made from specific ratios of stSEPS (sulfonated tSEPS)/PP blends have demonstrated promising hydrophilicity, fair mechanical characteristics, and platelet compatibility.

## 2. Materials and Methods 

### 2.1. Materials

Acetone (99%), argon gas (99.99%), and ethanol (95%) were used without further purifications. Calcium hydride (90–95%), 4-tert-butyl styrene (tBS) (99%), isoprene (99%), styrene (99%), and 1,2–Dichloromethane (≥99%) were purchased from Alfa Aesar, Tewksbury, MA, USA. Anhydrous methanol was obtained from MACRON, Allentown, PA, USA and n-butyl-lithium solution, 2.5M in hexane was from ALBEMARLE Charlotte, NC, USA, and tetrahydrofuran (THF) (99.9%) was from DUKSAN, Ansan-si Gyeonggi-do, Korea. The citric acid (99%) was purchased from SHOWA, Japan. The catalyst, cobalt 2-ethyl hexanoate solution (65 wt% in mineral spirits, mineral oil), acetic anhydride (≥99%), glutaraldehyde (50% in H2O), Hepes [*N*-(2-Hydroxyethyl-piperazine)-*N*’-(2-ethanesulfonic acid)] (≥99.5%), potassium chloride (≥99.5%), and magnesium chloride hexahydrate (98%) were obtained from Sigma Aldrich, St. Louis, MO, USA. D(+)-Glucose (≥99.5%) was from VETEC, St. Louis, MO, USA. Hydrogen gas was 99.9% from a local source and triethylaluminum, 1M in n-hexane was bought from KANTO, Japan. Hepes [*N*-(2-Hydroxyethyl-piperazine)-*N*’-(2-ethanesulfonic acid)] sodium salt (99.6%) was from J.T. Baker, Allentown, PA, USA. Hexamethyldisilizane (HMDS) (98+%) and sodium dihydrogen phosphate dihydrate (99%) were purchased from Alfa Aesar, Tewksbury, MA, USA. Mineral oil (hydrogenated) was obtained from Bioreagent, Sigma Aldrich, St. Louis, MO, USA. Platelet-rich plasma with CPDA-1 anticoagulant was obtained from the Tainan blood donation center. 

### 2.2. Synthesis of tBS-Styrene-Isoprene Block Copolymer (tSIS)

**Figure 1:**
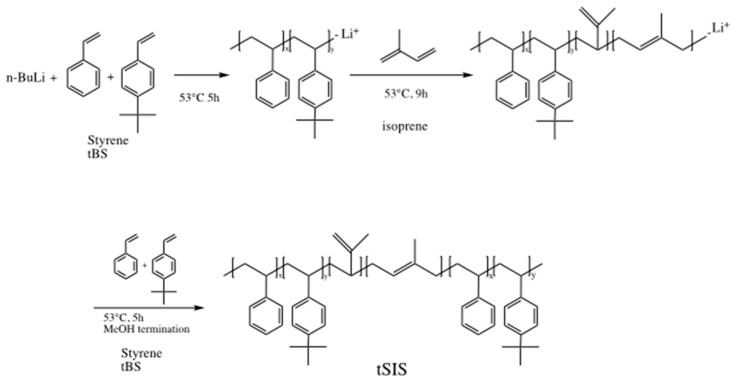
Synthesis scheme for tert-butyl styrene (tBS)-styrene-isoprene block copolymer (tSIS).

The synthesis scheme of the tSIS block copolymer was conducted in a dry argon atmosphere, and the reaction scheme is shown in Figure 1. Styrene, 4-tert-butyl styrene and isoprene were purified by distillation at a reduced pressure over calcium hydride and stored at −18 °C until use. Anionic polymerization was applied with styrene and tert-butyl styrene and isoprene as monomers in a cyclohexane solvent. *N*-butyl lithium was used as an initiator for the A–B–A elastomer synthesis. As the anionic polymerization was sensitive to oxygen and moisture, the pretreatment of monomers and solvents was very critical for the success of this reaction. In this study, tSIS was prepared based on the different weight ratio of styrene to 4-tert-butyl styrene: 5:5, 6:4, 7:3, 8:2, and 9:1. The weight ratio of isoprene to styrene/tert-butyl styrene was controlled at 5:2. The finished polymers were named as tSIS-5:5, tSIS-6:4, tSIS-7:3, tSIS-8:2, tSIS-9:1, respectively. The details are shown in Table 1.

### 2.3. Hydrogenation of tSIS Copolymer

**Figure 2:**
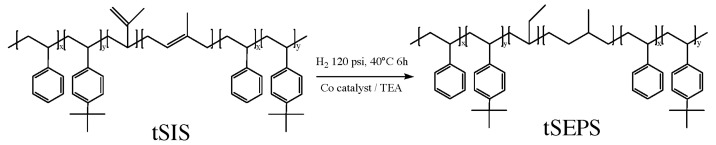
Reaction scheme for hydrogenation of tSIS copolymer.

The reaction scheme is shown in Figure 2. Following the previous studies [17,18,19,20,21], cyclohexane and 0.71 mL (1.34 mmol) cobalt (II) 2-ethyl hexanoate solution were purged with argon in a 100 mL flask for 30 min. Then, 4.8 mL (4.8 mmol) of triethyl-aluminum was added and the reaction was carried out under room temperature for 60 min. The catalyst solution became homogeneous and was ready to use when the color turned from dark blue to black. 

The high-pressure reaction vessel was purged with argon and then filled with 8 mL of catalyst solution and tSIS block copolymer solution (6 g in 100 mL cyclohexane) and the hydrogenation reaction proceeded under 120 psi and 40 °C for 6 h. The hydrogenated tSIS block copolymer, tSEPS (tert-butyl styrene/styrene-ethylene-propylene-tert-butyl styrene/styrene), product was then purified with 350 mL ethanol/acetone (*w/w* 1:1) solution for three times and then vacuum dried at 40 °C for 24 h. The residual catalysts were removed from the dried samples after extracting the samples/100 mL cyclohexane solution with 400 mL citric acid for three times. The organic-phase extract was then precipitated in 300 mL ethanol/acetone. The final tSEPS products were vacuumed dried at 40 °C for 24 h.

### 2.4. Preparation of Film Made by Blending Hydrogenated tSIS (tSEPS) with PP

In order to develop a proper blending ratio with good mixability for better uniformity and elongation, mineral oil was added as a compatibilizer, which was washed and eluted away afterward. Six mixing ratios were prepared according to Table 2. For blending experiments, the tSEPS used was prepared with styrene to tBS ratio fixed at 7:3. In a 50 mL flask, block copolymer and mineral oil were added and then heated up to 180 °C with a mechanical stir in an oil bath. Then, melted PP was added and mixed for 15 min to obtain the final mixture.

Hot press was adopted for film preparation. Two flat and clean metal plates with Teflon surface lining were heated up to 180 °C and then the blended mixture was placed into the space of two metal plates and pressed with 25 kg/m^2^ for 2 min. Then, the cooled pressed film was released from Teflon lining to get the final blended film. After mechanical blending, and hot-press process, uniform films with 100~250 μm were obtained.

### 2.5. Sulfonation of tSEPS/PP Film

Acetyl sulfate for the sulfonation of tSEPS/PP film was prepared as follows [22]: 39.6 mL CH_2_Cl_2_ and 7.6 mL acetic anhydride were added into a 100 mL flask in the ice bath. Then, 2.8 mL sulfuric acid was added under mechanical stirring for one hour.

**Figure 3:**
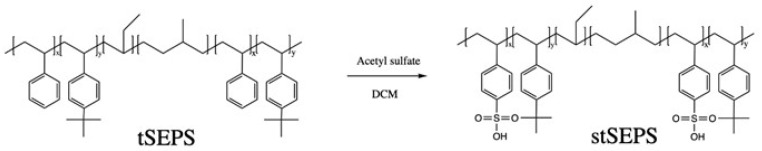
Reaction scheme of sulfonation of tSEPS.

A brief sulfonation scheme for the tSEPS is shown in Figure 3. In short, tSEPS/PP film was immersed in 75 mL dichloromethane in 100 mL flask under argon for 30 min. Then, the temperature was heated up to 50 °C, while 4.5 mL acetyl sulfate was added for the reaction for 15 min. Then, films were transferred to isopropyl alcohol to terminate the reaction. Finally, films were rinsed with double distilled water for several times until neutralization of water solution. The mineral oil used for preparing the tSEPS/PP film can be eluted by the immersion in dichloromethane before the sulfonation as well as by the dichloromethane in the acetyl sulfate/dichloromethane solution. Sulfonated polymer films (stSEPS/PP) were desiccated by a vacuum oven for 24 h at 40 °C prior to Attenuated Total Reflection-Fourier Transformed Infrared Spectroscopy (ATR-FTIR) and Electron Spectroscopy for Chemical Analysis (ESCA) analysis. It was noted that sulfonated tSEPS/PP-100% sample film was highly water soluble in which further investigation was not able to complete.

### 2.6. Water Uptake Analysis

Sulfonated polymeric film (stSEPS/PP) was cut into 1 cm × 1 cm size and then immersed in double-distilled water for 24 h. Samples were then removed with simple surface wiping to remove water residue and then the wet (swollen) weight (*M_wet_*) was determined. The dry weight (*M_dry_*) was taken right after samples were dried in a vacuum oven at 40 °C for 24 h. The water update (WU) ratio was calculated as follows:

$$\mathrm{WU}\left(\%\right)=\frac{M_{wet}-M_{dry}}{M_{dry}}\times 100\%$$

### 2.7. Thermal Gravimetric Analysis (TGA)

The weight of the sample film was about 3~10 mg, and the experiment was carried out in the range of 30 to 550 °C at the heating speed of 10 °C/min. The weight of each sample at different temperatures was then recorded.

### 2.8. Tensile Strength Analysis

The tensile strength of materials was conducted according to ASTM D882 standard by a Mechanical Testing System. The sample was cut to 70 mm × 5 mm size and 10 mm of each leading edge in the vertical direction was clamped and tensile strength and elongation were tested at 50 mm/min.

### 2.9. Platelet Adhesion Assay

Polymer sample films were trimmed to a size of 1 cm × 1 cm and placed into a 24-well plate sterilized with 75% ethanol 8 times on the orbital shaker at 100 rpm. Then, samples were immersed in a 10 mL Hepes-Tyrodes buffer solution for 1 h. Then, the buffer solution was removed and 10 mL of platelet-rich plasma was added into each well for incubation under 37 °C and 5% CO2 condition for 1 h. Then, platelet-rich plasma was replaced by 10 mL Hepes-Tyrodes buffer solution under 50 rpm shaking for 1 min 3 times. Then, the sample films were dehydrated by rinsing with 0%, 25%, 50%, 75%, and 100% ethanol subsequently for 1 min each, and later followed by complete dehydration with HMDS immersion for 5 min. Finally, HMDS was removed and dried before the sputter-coating with Au for the SEM examination.

## 3. Results

### 3.1. Characterization of tBS-Styrene-Isoprene Block Copolymer (tSIS) and Hydrogenated tSIS Copolymer (tSEPS)

Anionic polymerization was utilized for the synthesis of different ratios of Styrene/tert-butyl styrene with isoprene. The atomic ratio of each block was calculated by the area integration of the characteristic peak [23,24]. The chemical shift δ of each specific chemical peak was listed in Table S1. (Supplementary Table S1)

**Figure 4:**
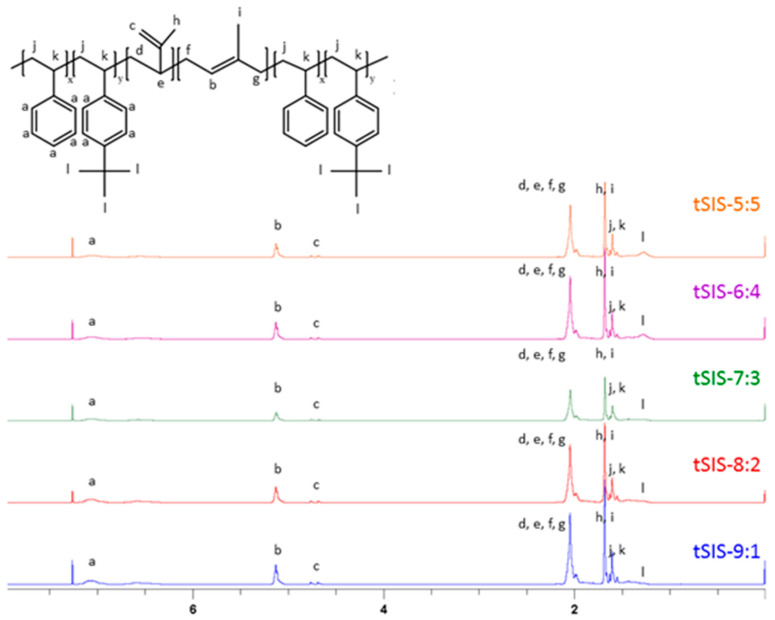
1H-NMR Spectrum of tSIS block copolymer with different ratio of styrene to tBS.

Based on Figure 4, the peak area of 1.3 ppm decreased with the increment of styrene ratio from 5:5 to 9:1. The specific proton chemical shifts demonstrated that the reaction was well controlled. The ratio of each block was calculated as below, and the result is shown in Table 3.

$$4-tert-\mathrm{Butylstyrene}=\frac{E}{9}\;\mathrm{Styrene}=\frac{A-4\times \frac{E}{9}}{5}\;\mathrm{Isoprene}=B+\frac{C}{2}$$

A denoted the protons of styrene, including five protons of styrene and four protons of tert-butyl styrene (area under peak a). B represented one unsaturated proton of 1,4-isoprene (area under peak b). C indicated two unsaturated protons of 3,4-isoprene (area under c). E was nine protons of the tert-butyl group (area under peak l).

The styrenic ratio represented the molar ratio of styrene/tBS to isoprene. In the experiment design, the weight ratio of styrene/tBS to isoprene was fixed at 2:5. After converting to the molar ratio, the theoretical values are close to 0.2, the experimental ones. Nevertheless, the molar ratio of styrene to tBS showed quite a difference between the theoretical and experimental ones, which likely resulted from the higher reactivity of tBS than styrene.

Yellowing is an issue that can restrict applications of tSIS due to unsaturated chemical bonding in tSIS. This became a hurdle during the sterilization of tSIS for biomedical applications. Therefore, the hydrogenation of tSIS was beneficial for improving thermal resistance capability. Several critical factors, such as catalyst, concentration, reaction time, and process temperature, can affect the degree of hydrogenation reaction.

All 1H-NMR spectra (Supplementary Figure S1) showed that the protons of unsaturated bonding on 1,4-isoprene and 3,4-isoprene (chemical shift δ: 4.5–5.2 ppm) completely vanished after hydrogenation treatment, while aromatic protons (chemical shift δ: 6.5–7.2 ppm) remained unchanged. This implied that the main saturation reaction is mainly on a soft isoprene block rather on the aromatic structure. Besides, the entire unsaturated bonding area was not detectable, meaning complete saturation after the hydrogenation reaction.

GPC analyses revealed the molecular weight of the tSIS-5:5, tSIS-6:4, tSIS-7:3, tSIS-8:2, and tSIS-9:1 is 118,146, 124,856, 103,543, 105,723, and 118,738, respectively. After hydrogenation, the molecular weight of the tSEPS-5:5, tSEPS-6:4, tSEPS-7:3, tSEPS-8:2, and tSEPS-9:1 has become 118,738, 118,849, 108,349, 111,432, and 102,319, respectively. The GPC results showed that, after hydrogenation, the molecule weight still remained in more than the 100k range. This suggested the polymer decomposition was not obvious, although hydrogenation was carried out under harsh conditions.

### 3.2. Characterization of Sulfonation of tSEPS/PP Film (stSEPS/PP)

#### 3.2.1. ATR-FTIR and ESCA Analysis

The film was prepared from blending the tSEPS, the hydrogenated tSIS-7:3, with PP at different weight ratios (Table 2). Several studies articulated that the sulfonation process was quite efficient to obtain a highly sulfonated film in 45 min [25,26]. Moreover, these studies have mentioned that chloro-sulfonic acid started to deteriorate mechanical strength by polymer chains scissoring in 5 min. Besides, as mentioned in the Materials and Methods section, tSEPS/PP-100% sample film was highly water-soluble after sulfonation. Therefore, a reaction time of 5/15/30 min was selected to determine the effectiveness of surface sulfonation by examining the surface characteristics of the sulfonated tSEPS/PP-70% with ATR-FTIR.

**Figure 5:**
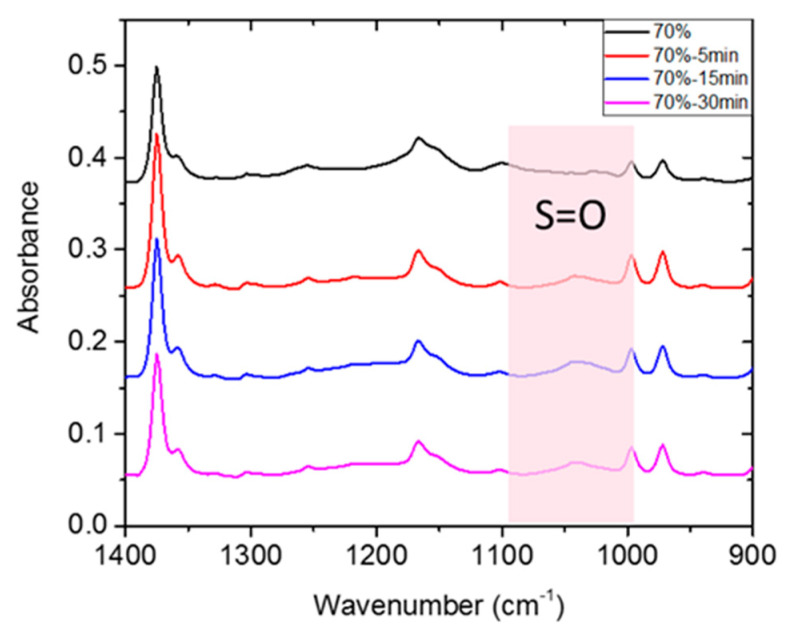
ATR-FTIR of sulfonated tSEPS/PP-70% under different sulfonation times.

A slightly symmetric stretching vibration of S=O on sulfonic groups was noticed around 1035 cm^−1^ in Figure 5. This suggested that surface sulfonation has occurred on all tSEPS/PP-70% specimens after different treatment duration. Nevertheless, as the sulfonation process could jeopardize the physical strength of composite films, 30 min of sulfonation of tSEPS/PP-70% film became fragile in water. Therefore, 15 min of sulfonation was dedicated to further experimental testing.

The surface chemical characteristics were also analyzed by the ESCA. The data given in Table 4 indicated that, after 15 min of sulfonation reaction, the O1s intensity increased from 2.65% to 8.69% with the increase in tSEPS content. The S2p atomic percentage was increased with the tSEPS content except the one prepared from 70% of tSEPS. A decrease in the S2p (%) of the stSEPS/PP-70% is likely due to the partial elution of stSEPS from the film to the dichloromethane if the film was prepared at a high percentage of tSEPS. For the sample prepared from the pure polypropylene (i.e., stSEPS/PP-0%), similar results had also been reached by Toda et al., in which the polypropylene tended to be sulfonated, as demonstrated by the presence of O1s and S2p signals [27]. 

The curve fitting results of the S2p peak are shown in Table 5 and Figure S2 in Supplementary Material. It was noted that the area ratio of characteristic S2p3/2 and S2p1/2 peaks was 2:1 for all samples, which was aligned with the theoretical value. This suggested the SO_3_/SO_2_ functionalities were formed after sulfonation.

#### 3.2.2. Surface Hydrophilicity of stSEPS/PP Film

**Figure 6:**
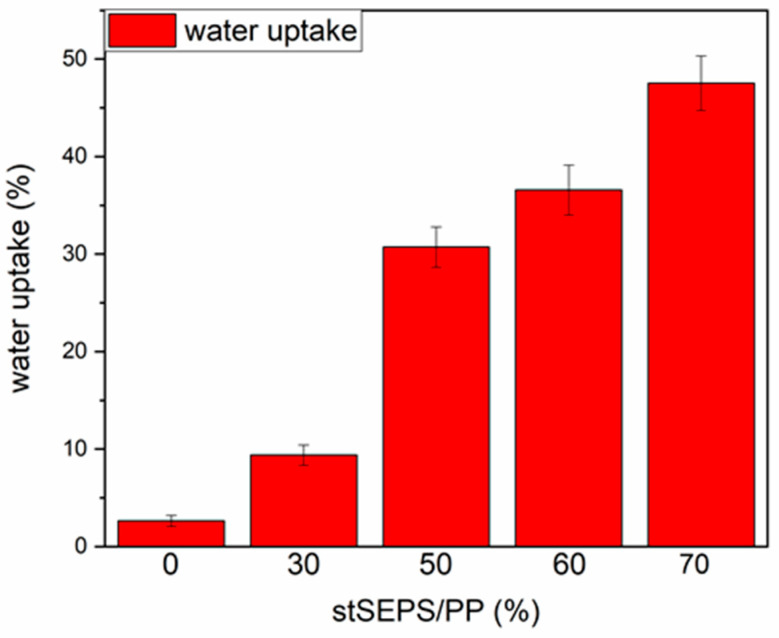
Water uptake of stSEPS/PP films at different blending ratios.

Figure 6 demonstrated the swelling ratio of different stSEPS/PP samples. The water uptake ratio increased with the weight percentage of tSEPS.

#### 3.2.3. Analysis of Thermal and Mechanical Properties

The thermal decomposition temperatures for different materials are shown in Table 6. It was noted that the saturated tSEPS had better thermal stability than unsaturated tSIS. The maximum thermal degradation temperature increased from about 400 to the 450 °C after the hydrogenation process. Besides, the sulfonated blended samples showed a higher thermal degradation temperature compared to those without sulfonation, except the pure PP (i.e., tSEPS% = 0). This can be attributed to the formation of the sulfonic bridge/sulfuric bridge, a linkage similar to the vulcanization, which led to the increase in thermal degradation temperature [28,29].

The tensile strength and the strain at break for different blending samples before and after the sulfonation are shown in Table 7 and Figure S3 in Supplementary Material. After blending with the PP, the tensile strength of tSEPS/PP samples increased while the strain at break decreased as compared to the neat tSEPS-7:3. Additionally, these changes were correlated to the weight percentage of tSEPS in the blending samples. After sulfonation, the tensile strength further increased and strain at break decreased for the samples with less than 60 wt% of tSEPS. This is likely due to the formation of the sulfonic bridge/sulfuric bridge, similar to the vulcanization, among these specimens. Nevertheless, for blends prepared from a higher weight percentage of tSEPS, the sulfonation could alter the intra- or inter-molecular arrangement of stSEPS/PP, resulting in a significant reduction in the strain at break with less significant variation in the tensile strength as compared to the non-sulfonated ones.

#### 3.2.4. Platelet Adhesion Testing on Non-Sulfonated and Sulfonated tSEPS/PP Films

**Figure 7:**
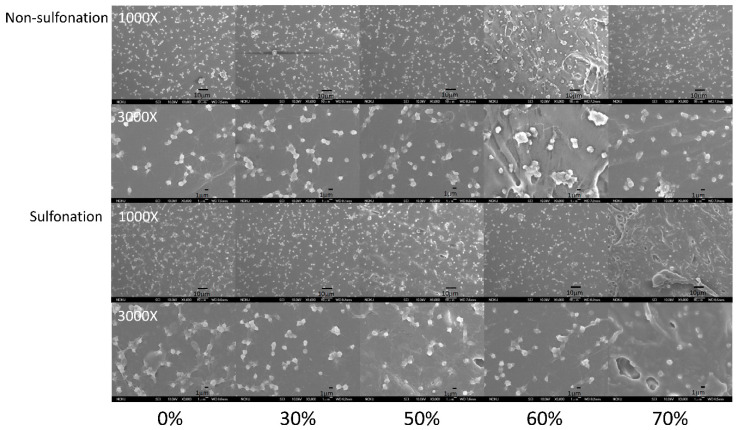
The SEM micrographs of the platelets adhered onto the blended tSEPS/PP at different blending ratio before and after surface sulfonation.

**Figure 8:**
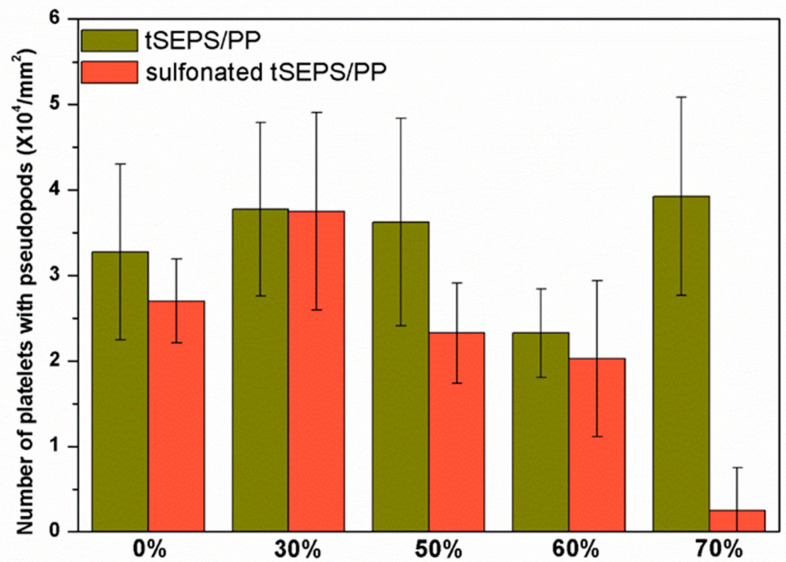
Platelet adhesion density on the blended tSEPS/PP at different blending ratios before and after surface sulfonation.

The platelet adhesion results on the non-sulfonated and sulfonated blending films are shown in Figure 7 and Figure 8. The SEM micrographs at 3000× magnification showed that the platelets with pseudopods and even aggregation were noted on the blended tSEPS/PP before sulfonation. Besides, the platelet adhesion density among these non-sulfonated samples was similar to each other except the one prepared with 60% of tSEPS (Figure 8). 

After sulfonation, the sulfonated tSEPS/PP-70% exhibited the least amount of platelet adherence. Besides, the degree of platelet activation and aggregation on the sulfonated tSEPS/PP-70% is less than the non-sulfonated one. This implicated the amount of water adsorbed in the sulfonated tSEPS/PP-70% (Figure 6) could take some roles, such as the formation of the water layer, in reducing the platelet adhesion and activation. 

## 4. Conclusions

Styrene-isoprene-styrene block copolymers with the tert-butyl group at different ratios were synthesized and hydrogenated successfully. By controlling the weight ratio of tert-butyl styrene in tSEPS, the sulfonation of films prepared by blending the hydrogenated copolymers (tSEPS) with PP has been demonstrated to have the hydrophilic swollen characteristics without losing its physical intact. Surface characterization by ESCA and ATR-FTIR analyses revealed the formation of SO_3_/SO_2_ functionalities after the sulfonation reaction. Platelet adhesion assay indicated that the sulfonation reaction for the tSEPS/PP blended films could effectively decrease the platelet adhesion and activation if the weight percentage of tSEPS is high. In addition, surface sulfonation has been shown to increase the decomposition temperature and the tensile strength while reducing the strain at break for the tSEPS/PP blended films. Further studies will be continued for optimizing the physical performance of stSEPS/PP blends as a potential alternative for medical storage applications.

## Figures and Tables

**Table 1:** Monomer weight ratio for tSIS block copolymer synthesis.

	Styrenic Block (g)		Isoprenic Block (g)	Styrenic Block (g)	
	Styrene	tBS	Isoprene	Styrene	tBS
tSIS5:5	1	1	10	1	1
tSIS6:4	1.2	0.8	10	1.2	0.8
tSIS7:3	1.4	0.6	10	1.4	0.6
tSIS8:2	1.6	0.4	10	1.6	0.4
tSIS9:1	1.8	0.2	10	1.8	0.2

**Table 2:** The blending ratio of tSEPS and PP.

	tSEPS (g)	PP (g)	Mineral Oil (g)	Total Weight (g)
tSEPS/PP-0%	0.0	3.0	0.3	3.3
tSEPS/PP-30%	0.9	2.1	0.3	3.3
tSEPS/PP-50%	1.5	1.5	0.3	3.3
tSEPS/PP-60%	1.8	1.2	0.3	3.3
tSEPS/PP-70%	2.1	0.9	0.3	3.3
tSEPS/PP-100%	3.0	0.0	0.3	3.3

tSEPS: tSEPS was the one prepared from hydrogenation of tSIS-7:3 (Styrene: tBS = 7:3).

**Table 3:** Block molar ratio of tSIS block copolymer.

	tSIS-5:5	tSIS-6:4	tSIS-7:3	tSIS-8:2	tSIS-9:1
Styrene: tBS (wt ratio)	5:5	6:4	7:3	8:2	9:1
Styrenic ratio (the*)	0.18	0.18	0.19	0.20	0.20
Styrenic ratio (exp*)	0.19	0.19	0.22	0.22	0.22
Styrene: tBS (the*)	1.54	2.31	3.59	6.15	13.85
Styrene: tBS (exp*)	0.56	0.83	1.63	2.00	2.51

the*: theoretical molar ratio; exp*: experimental molar ratio.

**Table 4:** Atomic ratio of stSEPS/PP copolymer films.

	C1s (%)	O1s (%)	S2p (%)
**stSEPS/PP-0%**	96.38	3.27	0.36
**stSEPS/PP-30%**	97.06	2.65	0.29
**stSEPS/PP-50%**	95.28	4.02	0.70
**stSEPS/PP-60%**	91.90	7.30	0.80
**stSEPS/PP-70%**	91.08	8.69	0.23

**Table 5:** Area percentage of S2p peak in stSEPS/PP copolymer films.

	SO_3_ 2p_1/2_	SO_3_ 2p_3/2_	SO_2_ 2p_1/2_	SO_2_ 2p_3/2_
	171.2 eV	170.0 eV	170.3 eV	169.1 eV
**stSEPS/PP-0%**	22.18	44.36	11.15	22.30
**stSEPS/PP-30%**	23.53	47.05	9.81	19.62
**stSEPS/PP-50%**	29.58	59.15	3.76	7.51
**stSEPS/PP-60%**	29.81	59.62	3.52	7.04
**stSEPS/PP-70%**	26.51	53.02	6.82	13.64

**Table 6:** Thermal decomposition temperature for different specimen.

Styrene: tBS	T_dmax_ (°C)		tSEPS ^#^ (%)	T_dmax_ (°C)	
	tSIS	tSEPS		tSEPS/PP	stSEPS/PP
**5:5**	393.18	452.44	**0%**	438.22	432.73
**6:4**	391.15	448.52	**30%**	452.45	453.44
**7:3**	381.86	441.73	**50%**	435.26	444.19
**8:2**	391.59	440.63	**60%**	435.23	446.27
**9:1**	384.96	449.63	**70%**	447.13	457.02

#: tSEPS used for blending with PP is prepared from tSEPS-7:3.

**Table 7:** The stress–strain analysis for different specimen (n = 3).

	Tensile Strength (MPa)	Strain at Break (%)
**tSEPS-7:3**	0.46 ± 0.06	134.65 ± 5.97
**tSEPS ^#^/PP-0%**	16.87 ± 1.21	4.99 ± 0.68
**tSEPS/PP-30%**	8.13 ± 0.36	9.32 ± 0.95
**tSEPS/PP-50%**	5.11 ± 0.29	18.89 ± 5.63
**tSEPS/PP-60%**	2.78 ± 0.12	27.96 ± 0.93
**tSEPS/PP-70%**	1.08 ± 0.00	43.83 ± 4.17
**stSEPS/PP-0%**	22.45 ± 0.55	3.83 ± 0.24
**stSEPS/PP-30%**	13.19 ± 1.09	6.94 ± 0.87
**stSEPS/PP-50%**	7.21 ± 0.38	12.31 ± 0.44
**stSEPS/PP-60%**	2.06 ± 0.41	6.38 ± 0.87
**stSEPS/PP-70%**	1.15 ± 0.56	6.97 ± 0.86

#: tSEPS used for blending with PP is prepared from tSEPS-7:3.

## Supplementary Materials

The following are available online at https://www.mdpi.com/2073-4360/12/6/1351/s1, Figure S1: 1H-NMR Spectrum of tSIS and tSEPS block copolymer with different ratio of styrene to tBS, Figure S2: The S2p spectra in stSEPS/PP copolymer films, Figure S3: The stress-strain analysis for different specimen, Table S1: Proton chemical shift δ of tSIS.
