# Supplementary material for: Sulfonation and Characterization of Tert-Butyl Styrene/Styrene/Isoprene Copolymer and Polypropylene Blends for Blood Compatibility Applications
Source: Polymers (Basel). 2020 Jun 15;12(6):1351. doi: 10.3390/polym12061351 (PMC7361970; doi:10.3390/polym12061351)
Supplement: Supplementary file 1 [file polymers-12-01351-s001.pdf]

Supplementary materials

Table S1 Proton chemical shift δ of tSIS

6.5-7.2 ppm	peak a; assigned to aromatic proton on styrene
5.0-5.2 ppm	peak b; assigned to the olefinic proton of isoprene units in 1,4 addition
4.5-4.8 ppm	peak c; assigned to the olefinic proton of isoprene units in 3,4 addition
1.8-2.2 ppm	peak d-g; assigned to the proton of–CH_2– and >CH– of isoprene
1.6-1.8 ppm	peak h-k; assigned to saturated protons of the methyl group of 1, 4 isoprene and 3, 4 isoprene. Protons of –CH_2– and >CH– of the aromatic ring of styrene
1.3 ppm	peak l; assigned to the proton of butyl group of tBS

Fig. S1

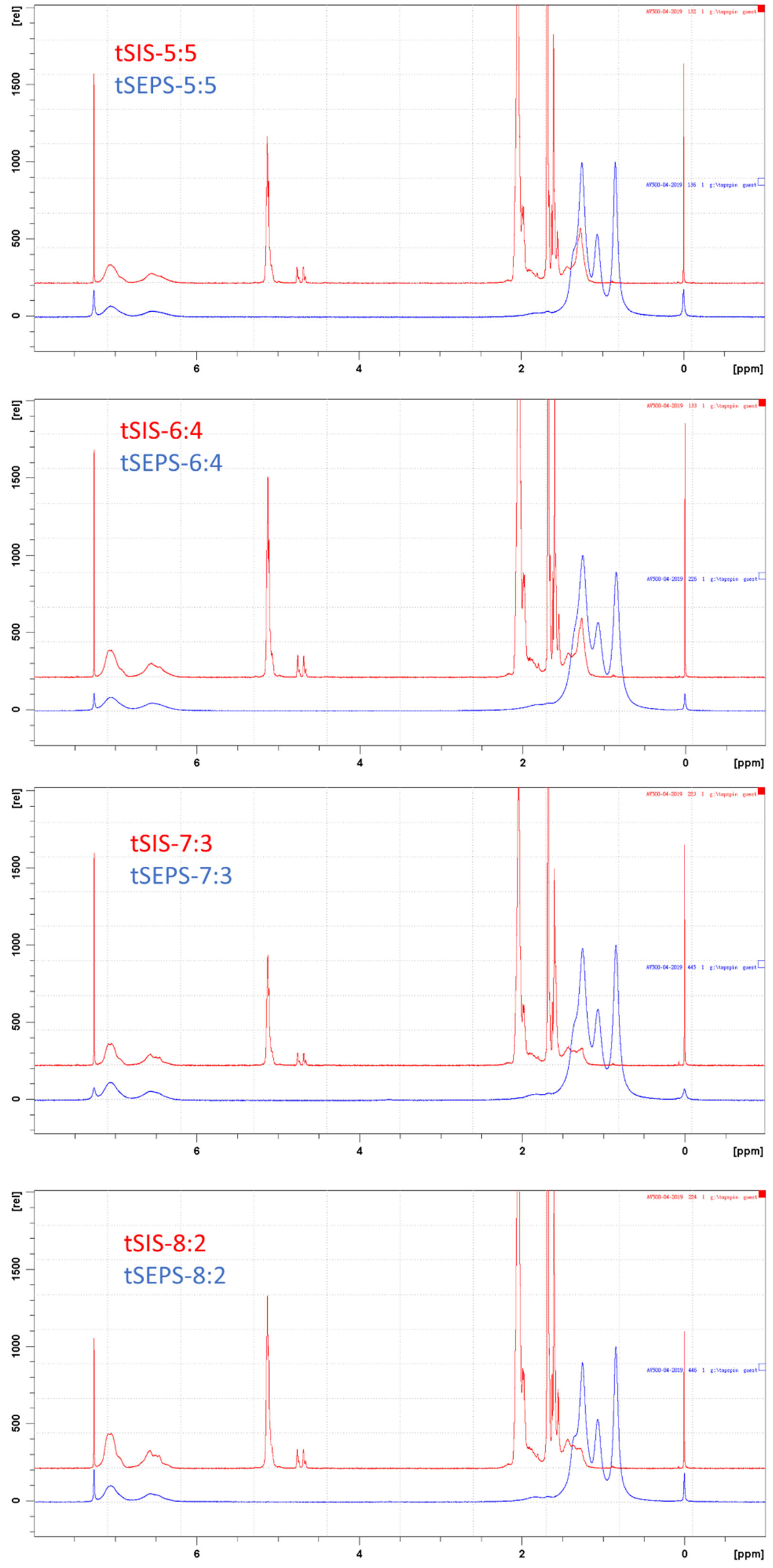
tSIS-5:5
tSEPS-5:5
tSIS-6:4
tSEPS-6:4
tSIS-7:3
tSEPS-7:3
tSIS-8:2
tSEPS-8:2
[rel]
[ppm]

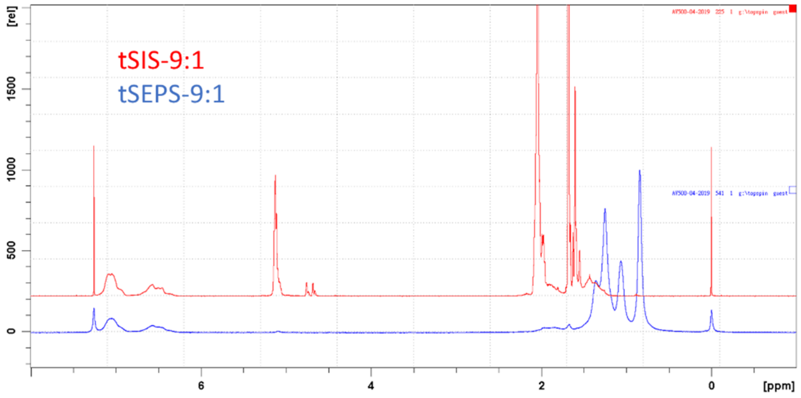

Figure S1. 1H-NMR Spectrum of tSIS and tSEPS block copolymer with different ratio of styrene to tBS

Fig. S2

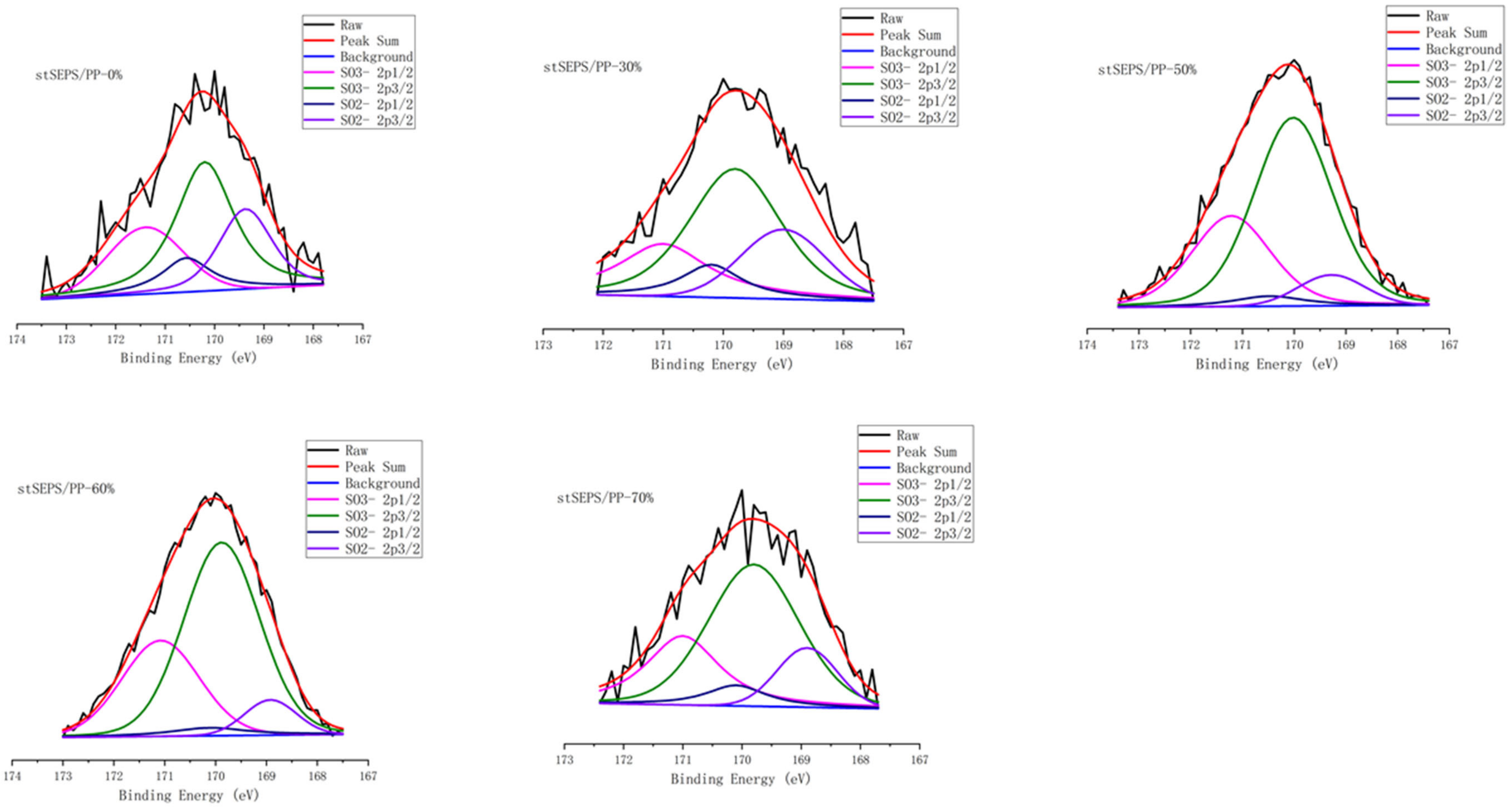

Figure S2. The S2p spectra in stSEPS/PP copolymer films

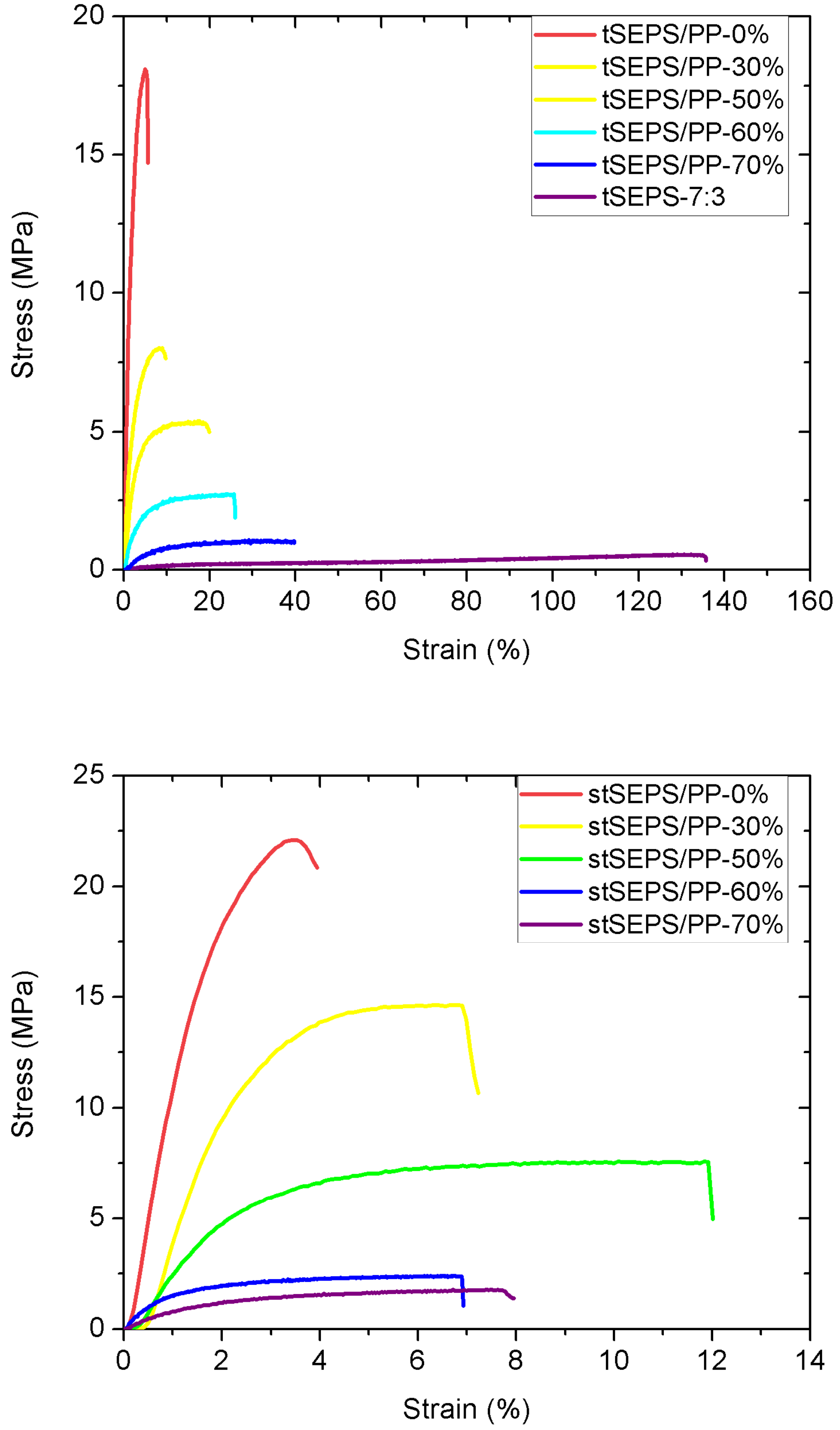

Figure S3. The stress-strain analysis for different specimen
